# Competition between Serum IgG, IgM, and IgA Anti-Glycan Antibodies

**DOI:** 10.1371/journal.pone.0119298

**Published:** 2015-03-25

**Authors:** Saddam M. Muthana, Li Xia, Christopher T. Campbell, Yalong Zhang, Jeffrey C. Gildersleeve

**Affiliations:** Chemical Biology Laboratory, National Cancer Institute, NIH, 376 Boyles St., Frederick, MD, 21702, United States of America; King's College London, UNITED KINGDOM

## Abstract

Anti-glycan antibodies are an abundant subpopulation of serum antibodies with critical functions in many immune processes. Changes in the levels of these antibodies can occur with the onset of disease, exposure to pathogens, or vaccination. As a result, there has been significant interest in exploiting anti-glycan antibodies as biomarkers for many diseases. Serum contains a mixture of anti-glycan antibodies that can recognize the same antigen, and competition for binding can potentially influence the detection of antibody subpopulations that are more relevant to disease processes. The most abundant antibody isotypes in serum are IgG, IgM, and IgA, but little is known regarding how these different isotypes compete for the same glycan antigen. In this study, we developed a multiplexed glycan microarray assay and applied it to evaluate how different isotypes of anti-glycan antibodies (IgA, IgG, and IgM) compete for printed glycan antigens. While IgG and IgA antibodies typically outcompete IgM for peptide or protein antigens, we found that IgM outcompete IgG and IgA for many glycan antigens. To illustrate the importance of this effect, we provide evidence that IgM competition can account for the unexpected observation that IgG of certain antigen specificities appear to be preferentially transported from mothers to fetuses. We demonstrate that IgM in maternal sera compete with IgG resulting in lower than expected IgG signals. Since cord blood contains very low levels of IgM, competition only affects maternal IgG signals, making it appear as though certain IgG antibodies are higher in cord blood than matched maternal blood. Taken together, the results highlight the importance of competition for studies involving anti-glycan antibodies.

## Introduction

Human serum contains a vast assortment of carbohydrate-binding antibodies that play critical roles in human health and provides a rich reservoir of potential biomarkers for many biomedical applications and diseases. For example, detection of anti-glycan antibodies against blood group A and B antigens provides a simple and reliable strategy to predict which individuals are suitable matches for transfusions and transplants [1–3]. Anti-glycan antibodies are also crucial in other areas of immunology, such as tumor surveillance, autoimmunity, defense against pathogens, and response to vaccines [4–7]. These broader immune functions have stimulated interest into exploring the potential use of circulating anti-glycan antibodies as biomarkers for a wide variety of diseases [8–17].

Detection of serum anti-glycan antibodies is typically carried out by immobilizing a carbohydrate of interest, capturing specific antibodies, and then measuring amounts of bound antibodies. This process is complicated by the fact that serum often contains a mixture of antibodies that recognize the same antigen, and certain antibodies within the mixture may be more relevant to immune protection or disease processes than others. Antibodies to a particular glycan may vary in terms of affinity, specificity, concentration, and/or isotype, but all can compete for binding to the same antigen. As a result, binding of one can influence detection of the others, and it can be challenging to reliably measure a target antibody subpopulation of interest.

One of the best examples illustrating the complexities of competition is detection of one antigen-specific antibody isotype in the presence of others. Different isotypes have distinct biological functions, and detection of different isotypes to a given antigen is critical for understanding the immune response and developing diagnostics. The most abundant antibody isotypes in serum are IgG, IgM, and IgA. Due to the affinity maturation and class switching processes, IgG and IgA antibodies typically have substantially higher affinities than IgM antibodies [18,19]. Consequently, IgG antibodies often outcompete IgM antibodies for antigens, making it difficult to reliably measure IgM levels. Additionally, rheumatoid factors (RF, antibodies that bind to human gamma immunoglobulins) can interfere with the measurement of IgM antibodies [20,21]. To address this, IgG antibodies are frequently removed prior to assaying for IgM antibodies to avoid false negative results that arise when high affinity IgG antibodies outcompete IgMs for binding sites, or to avoid false positive results that can arise when detecting IgM-class RF.

Carbohydrates are a unique class of antigens with unusual molecular recognition features. Unlike protein antigen, monovalent interactions between a single binding site of an antibody and a single glycan are generally weak. Even for affinity-matured IgG antibodies, monovalent binding dissociation constants for glycans are typically in the range of 100 nM to 100 μM. As a result, avidity effects play a more important role in achieving tight binding. Serum IgM antibodies are pentameric and have 10 binding sites whereas serum IgG and IgA are mainly monomeric with 2 binding sites. With more binding sites, carbohydrate-binding IgM have the potential to outcompete IgG and IgA through enhanced avidity. For the vast majority of serum anti-glycan antibodies, it is not known whether IgG, IgA, or IgM antibodies have a competitive advantage, or if the competitive advantage varies from one antigen-antibody pair to another.

Glycan array technology provides a powerful high-throughput tool for studying the interactions between carbohydrates and macromolecules, and to identify anti-glycan antibodies as biomarkers for variety of diseases [22–31]. It allows one to profile serum antibody levels for hundreds of glycans in a single experiment using minimal amounts of precious clinical samples and expensive or scarce carbohydrates. Therefore, glycan arrays provide the unique opportunity to evaluate antibody competition for numerous carbohydrate antigens in parallel. Herein, we used a multiplexed glycan array assay to evaluate how different isotypes of anti-glycan antibodies (IgA, IgG, and IgM) compete for the printed antigens.

## Results

Our approach for evaluating competition involved the use of purified IgG, IgA, and IgM antibodies from pooled human serum. Each polyclonal antibody sample would be profiled on our glycan microarray individually and in the presence of other isotypes. Additionally, changes in IgG and IgM anti-glycan antibody signals would be evaluated in whole serum upon addition of IgG, IgA, and IgM. Although IgD and IgE are also present in serum at low concentrations and capable of competing, this study focused on the highest abundance antibodies in serum.

### Selection of profiling parameters

The results of a competition experiment are dependent on the conditions used for the assay. One factor that could influence the outcome is the selection and quality of secondary reagents used to detect each antibody isotype. We evaluated the detection secondary antibodies to confirm their specificities and validate their use in developing a multiplexed microarray-based assay (see S1 File). From the SDS-PAGE gels and western blots of purified antibodies from human pooled serum (IgA, IgG, and IgM), we confirmed that the secondary antibodies have no cross-reactivity with other isotypes (Fig. A, S1 File). This experiment also confirmed that there are no detectable contaminating antibodies in the commercially-available purified isotype samples. Additionally, we verified that the IgG secondary reagent detects all the different IgG subclasses (IgG_1_-IgG_4_) (Fig. B, S1 File).

Next, we compared relative signal intensities for the secondary reagents. Different concentrations (25μg/mL to 800μg/mL) of purified serum antibodies (IgA, IgG, and IgM) were spotted onto nitrocellulose membranes and were detected by their corresponding secondary antibodies. The relative signals obtained for the IgM secondary were 20–30% and 45–55% higher than that of IgG and IgA, respectively (Figs. C and D, S1 File). Thus, raw signals obtained with each of the fluorophore-labeled secondaries at the same laser power and photomultiplier tube (PMT) voltage settings are fairly similar (within 2 fold) for equivalent amounts of IgG, IgA, or IgM. Finally, we validated the use of multiple secondary reagents in a single experiment to maximize throughput. DyLight 549 or DyLight 649-labeled secondary antibodies were found to have almost no bleed-through across the red and green channels (see Fig. E, S1 File). Data collected using a single secondary antibody was nearly identical with data collected in the presence of both secondary reagents (Fig. F, S1 File). Therefore, we can reliably detect and measure two different antibody isotypes in a single experiment.

The assay conditions could also influence the results. We typically evaluate human antibody profiles at 37°C. While binding at this temperature could potentially miss cold agglutinin antibodies that are present but only bind at lower temperatures, 37°C was selected to most accurately reflect binding under physiological conditions.

The concentrations of antibodies used in the competition can have a major impact on the results. The normal distribution of IgG, IgM, and IgA in the sera of healthy adults have been well studied [mean (95% range): IgG 9.9 (6.4–13.5) mg/mL, IgA 1.7 (0.7–3.1) mg/mL, and IgM 1.6 (0.6–3.5) mg/mL][32]. For the comparison, we opted to evaluate each purified antibody fraction at a concentration that was approximately similar to a serum dilution of 1:50 (200 μg/mL for IgG, 50 μg/mL for IgA, and 50 μg/mL for IgM), our standard dilution for evaluating human serum samples. At this concentration, many anti-glycan antibodies can be detected but relatively few signals are saturated. Although the exact proportion of anti-glycan antibody in each isotype fraction is unknown, this would allow us to evaluate the effects of changes relative to typical serum abundance. For example, addition of 200 μg/mL of IgG to 50 μg/mL of IgA would represent an addition of one “serum equivalent” of IgG (a “serum equivalent” refers to the concentration of antibody that would typically be found in serum at our working dilution of 1:50). The distribution and magnitude of signals obtained with purified IgG and IgM were similar to those obtained with a standard sample of pooled human serum (referred to as our “reference” sample), whereas purified IgA signals were lower than those found in our reference serum sample (Fig. G, S1 File).

### Profiles of individual isotypes

Purified IgG, IgA, and IgM were first profiled individually at various concentrations in the absence of other isotypes. IgM provided high signals to numerous glycans on the array (for full data, see S2 File). For example, at 50 μg/mL IgM there were 237 IgM signals that were at least 4-fold above the floor (a floor value of 150 RFU was used, which is approximately 2-fold over the background; all signals below this level were set to 150). Many IgG and IgA signals could also be detected, albeit at lower frequency than IgM. For example, at 200 μg/mL IgG there were 136 signals that were at least 4-fold above the floor, whereas 50 μg/mL IgA yielded only about 40 signals that were at least 4-fold above the floor. As expected, signals for all three isotypes increased with rising concentrations of antibody in an approximately linear fashion until saturated. To evaluate changes in signals in the sections below, we only considered glycans with signals that are at least 4-fold above floor value, such that we could detect a decrease in signal.

### Effects of IgM and IgA on IgG signals

**Fig 1:**
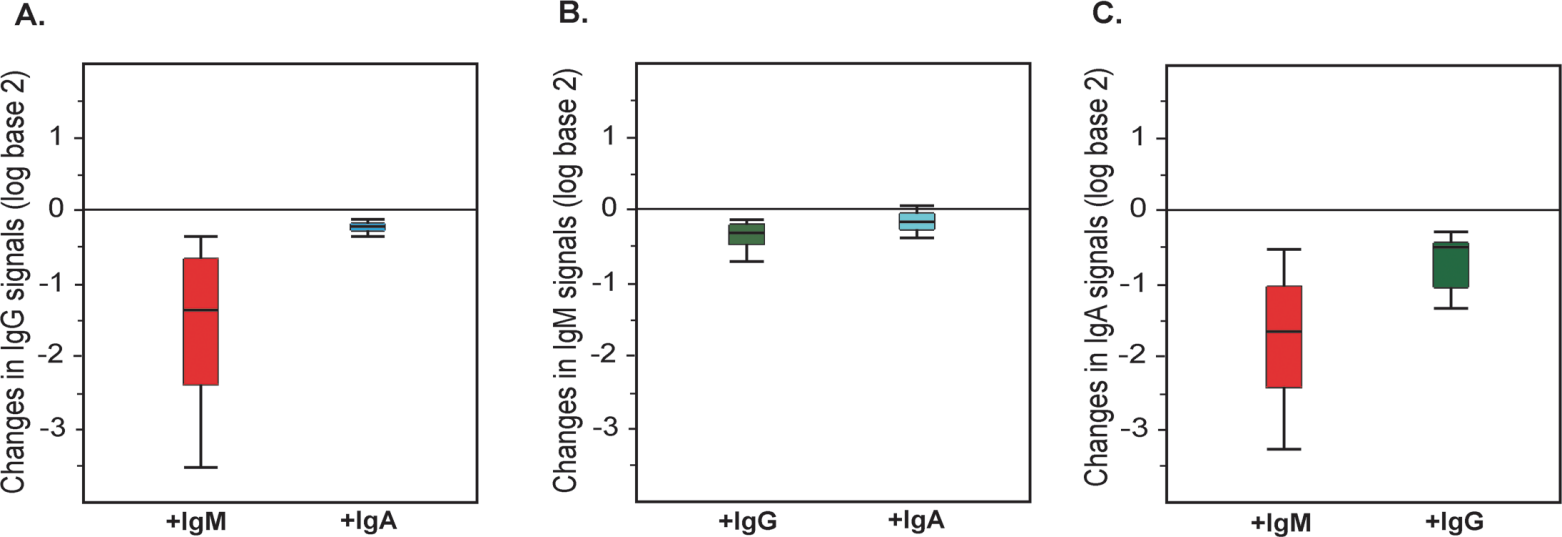
Competition between serum IgG, IgA, and IgM anti-glycan antibodies. (A) Addition of IgM and IgA to IgG. Polyclonal IgG isolated from serum was first profiled on the array alone. Separately, IgG was premixed with varying amounts of IgM or IgA and then profiled on the array. For each array component, the change in IgG signal in the presence of IgM or IgA was determined. The box plots depict the range of IgG changes (on a log base 2 scale) measured on the array upon addition of 4 serum equivalents of IgM or IgA. The line in the middle of the box is the median, the box spans 1 standard deviation above and below the median, and the whiskers represent 2 standard deviations above or below the median. (B) Addition of IgG and IgA to IgM. An analogous protocol as above was used to evaluate effects of IgG and IgA on IgM signals. (C) Addition of IgM and IgG to IgA. An analogous protocol as above was used to evaluate effects of IgG and IgM on IgA signals. The box plots demonstrate significant decreases in IgG and IgA signals in the presence of IgM for the vast majority of array components.

**Fig 2:**
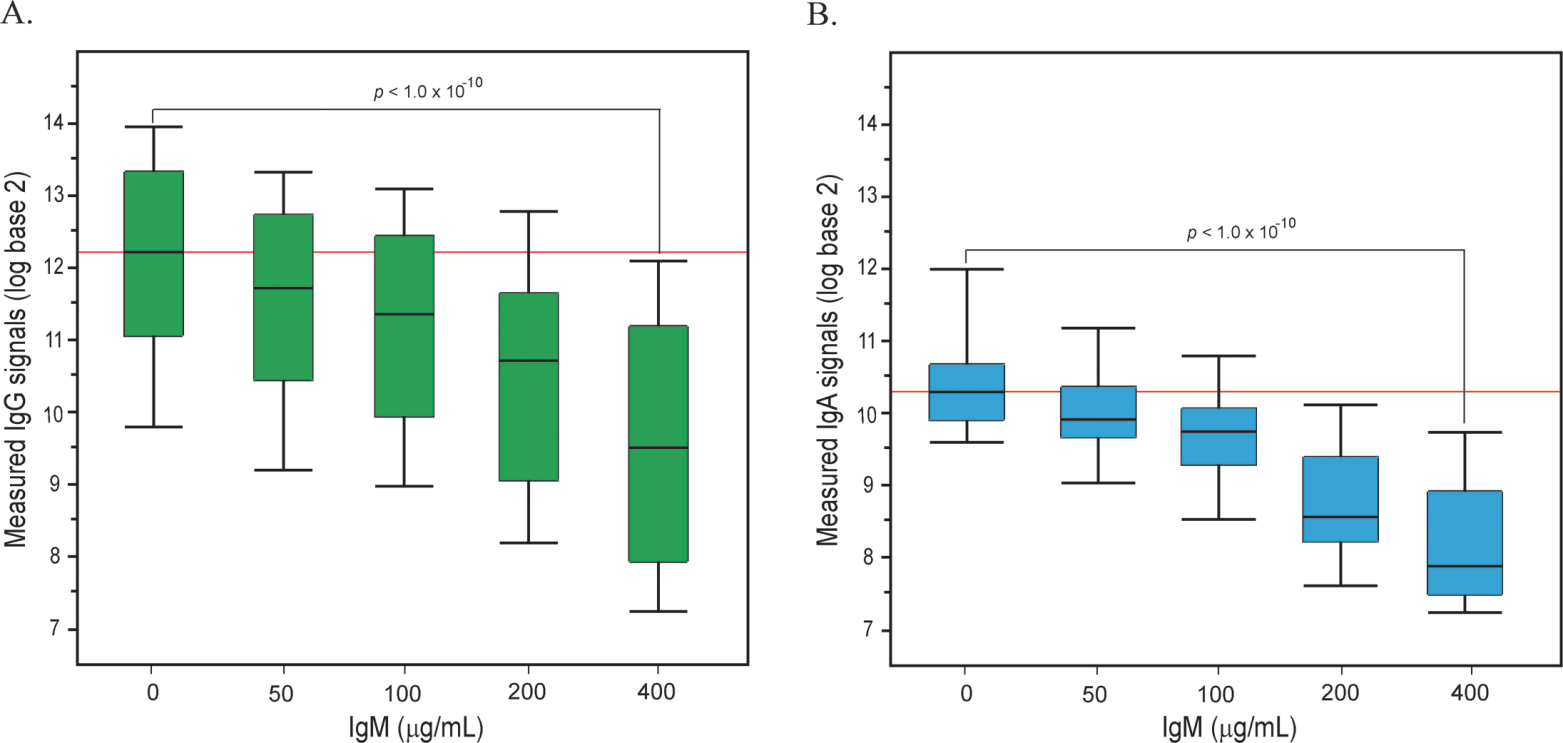
Dose-dependent inhibition of IgG and IgA signals upon addition of IgM. (A) Decrease in IgG signals with increase in IgM concentration. (B) Decrease in IgA signals with increase in IgM concentration. Polyclonal IgG and IgA isolated from serum were first profiled on the array alone. Separately, they were pre-mixed with varying amounts of IgM and then profiled on the array. The box plots depict the range of IgG or IgA signals (on a log base 2 scale) alone or in the presence of varying amounts of IgM (50–400 μg/mL). The line in the middle of the box is the median signal, the box spans 1 standard deviation above and below the median, and the whiskers represent 2 standard deviations above or below the median. The box plots demonstrate decreasing IgG and IgA signals in the presence of increasing amounts of IgM across the entire array.

**Fig 3:**
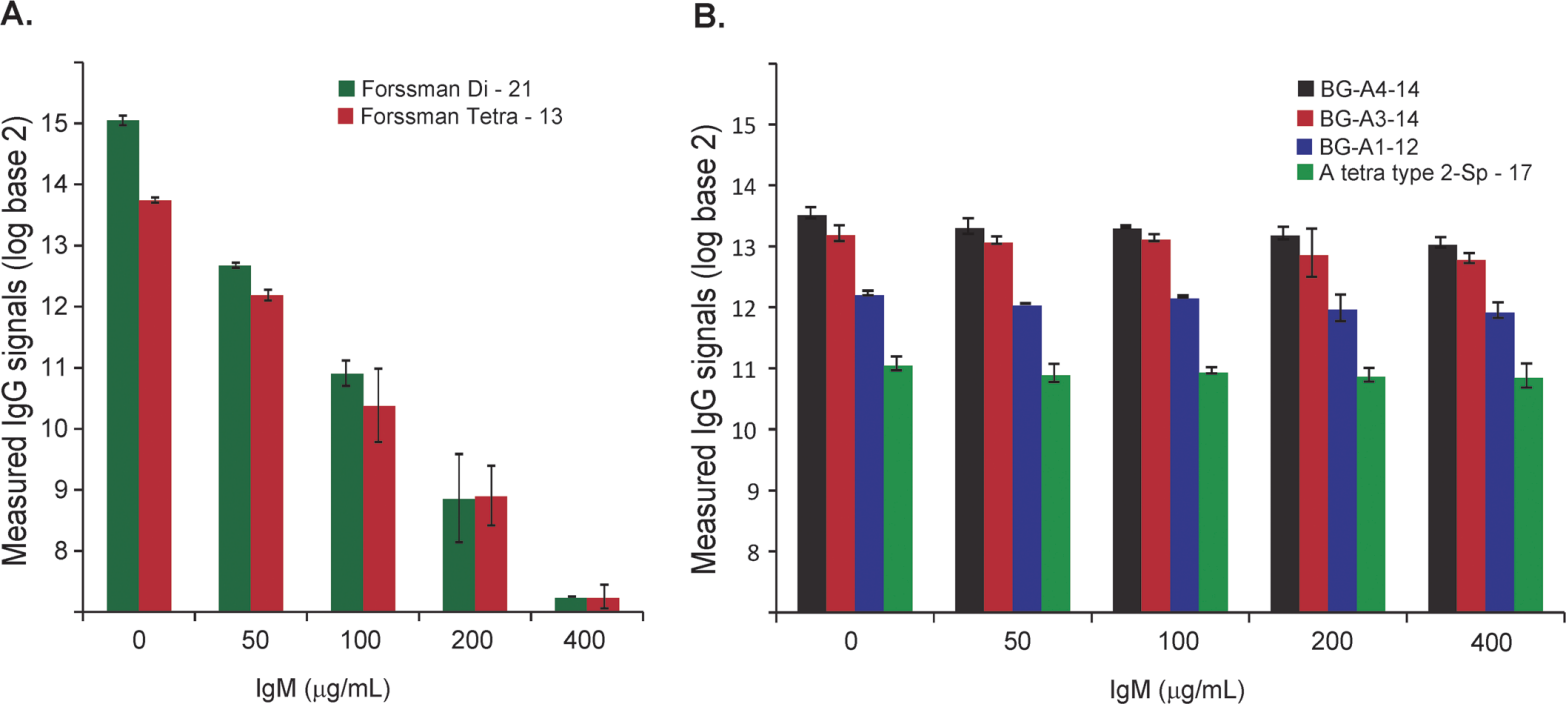
Dependence of inhibition on carbohydrate antigen structure. Measured IgG signals to Forssman disaccharide and tetrasaccharide (A) and four blood group A antigens (B) in the absence of added IgM (0 μg/mL) or in the presence of varying amounts of IgM (50–400 μg/mL).

Purified IgG was profiled at 200 μg/mL (1 “serum equivalent”) in presence of varying amounts of added IgM or IgA. IgG signals decreased significantly (*p* < 1.0 x 10^−15^) in the presence of 4 serum equivalents of IgM (200 μg/mL; Fig. 1A). The median drop in IgG signal was about 3-fold, and 84 signals (62%) decreased by more than 2-fold including 17 signals that dropped by 10-fold or greater (see Fig. H, S1 File). Varying the amount of added IgM resulted in a dose-dependent inhibition of anti-glycan IgG (Fig. 2A). The IgG signals dropped below our detection level for certain glycans such as Forssman antigens (GalNAcα1–3GalNAcβ1–3Galα1–4Galβ), GlcNAcβ, and some sialylated Lewis C antigens (LeC: Galβ1–3GlcNAcβ) in the presence of 400 μg/mL of IgM. In the case of the Forssman antibodies, this represents a 100-fold decrease in IgG signal. Addition of 400 μg/mL of IgM is analogous to a patient or subject having an 8-fold increase in serum IgM. Thus, a relatively modest increase in IgM to any of these glycans can greatly diminish or even prevent detection of IgG antibodies to these same glycans. It is important to note that the extent of competition varies for different glycans. For example, significant decreases in IgG signals to Forssman antigens can be achieve upon addition of only 50 μg/mL of IgM (Fig. 3A), but the addition of as much as 400 μg/mL of IgM had little effect on measured IgG signals to blood group A antigens (Fig. 3B). In contrast to IgM, added IgA had minimal effect on IgG signals (Fig. 1A). The changes in IgG signals were not statistically significant (*p* > 0.2) upon adding 200 μg/mL of IgA. Even with the addition of 400 μg/mL of IgA, only 2 IgG signals (1.5%) decreased by about 2-fold.

### Effects of IgG and IgA on IgM signals

Purified IgM was profiled at 50 μg/mL (1 “serum equivalent”) in presence of varying amounts of added IgG or IgA. Interestingly, addition of IgG or IgA had little or no effect on the IgM signals (Fig. 1B). For example, even with the addition of 800 μg/mL of IgG (4 “serum equivalents”), only 6 IgM signals (2.5%) decreased and the magnitude of those decreases was only about 2-fold (see Fig. I, S1 File). Likewise, addition of IgA also had little or no effect on IgM profiles. The changes in IgM signals in the presence of 4 “serum equivalents” (200 μg/mL) of IgA were not significant (*p* > 0.3). Upon the addition of 400 μg/mL IgA, 6 IgM signals (2.5%) decreased by about 2-fold.

It is important to note that the data showed no evidence for interference by rheumatoid factors (RF) for our samples. If IgM-class RF are present, one can observe false positives (cases where there is little or no anti-glycan IgM but an IgM signal is observed due to IgM-class RF binding to anti-glycan IgG) or general increases in IgM signal upon addition of IgG due to the detection of both anti-glycan IgM and IgM-class RF that bind anti-glycan IgGs. The measured IgM signals were the same in the absence or presence of IgG or IgA antibodies, indicating that our purified IgM sample does not contain IgM-class RF. Moreover, none of the glycans gave rise to IgM signals when profiling purified IgG, indicating the absence of IgM-class RF in the purified IgG sample.

### Effects of IgM and IgG on IgA signals

Purified IgA was profiled at 50 μg/mL (1 “serum equivalent”) in presence of varying amounts of added IgG or IgM. Similar to IgG, IgA signals decreased significantly (*p* < 1.0 x 10^−9^) in the presence of 4 serum equivalent of IgM (Fig. 1C). The median drop in IgA signals was over 3-fold in the presence of 200 μg/mL IgM, and 71% of all the IgA signals that were at least 4-fold above the floor decreased by 2-fold or greater (see Fig. J, S1 File). Varying the amount of added IgM resulted in a dose-dependent inhibition of anti-glycan IgA (Fig. 2B). The IgA signals dropped below our detection level for certain glycans including Forssman antigens, GlcNAcβ, and sialylated Lewis antigens upon the addition of 400 μg/mL of IgM. Like IgG, the results demonstrate that a relatively modest increase in serum IgM to any of these glycans can greatly diminish or even prevent detection of IgA antibodies to these same glycans. Addition of 4 serum equivalent of IgG also inhibited IgA binding (*p* = 0.004) but to a lesser degree than IgM. Upon addition of four “serum equivalents” (800 μg/mL) of IgG, about 1/4 of all the IgA signals that were at least 4-fold above the floor decreased by 2-fold or greater (Fig. J, S1 File). Thus, both IgM and IgG antibodies can competitively inhibit IgA signals.

### Effects upon addition of purified antibodies to serum

**Fig 4:**
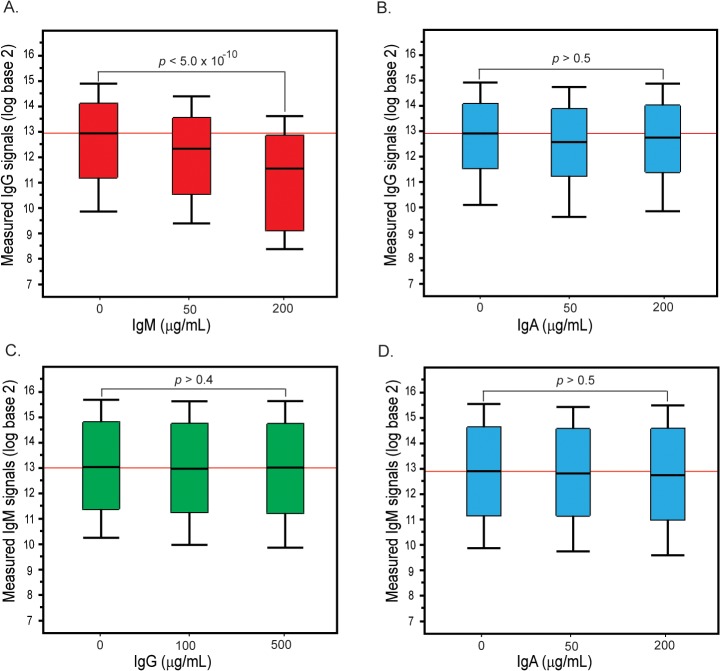
Changes in serum IgG and IgM signals in a pooled serum sample in the presence of varying amounts of added IgG, IgA, or IgM. (A) Changes in IgG signals upon addition of IgM. (B) Changes in IgG signals upon addition of IgA. (C) Changes in IgM signals upon addition of IgG. (D) Changes in IgM signals upon addition of IgA. IgG and IgM signals in a pooled serum sample were first profiled on the array alone. Separately, the serum sample was pre-mixed with varying amounts of IgG, IgA, or IgM and then profiled on the array. The box plots depict the range of IgG or IgM signals (on a log base 2 scale) alone or in the presence of varying amounts of IgG, IgA, or IgM. The line in the middle of the box is the median signal, the box spans 1 standard deviation above and below the median, and the whiskers represent 2 standard deviations above or below the median. The only significant decreases observed are for IgG signals upon addition of IgM.

Considering the findings that IgM anti-glycan antibodies appeared to inhibit IgG and IgA reactivity toward printed glycan antigens, we sought to verify these changes in pooled reference serum sample. We profiled IgG and IgM anti-glycan antibodies in our “reference” serum (1:50) in the presence or absence of varying amounts of added IgG, IgA, or IgM. As illustrated in Fig. 4, IgG signals in the reference sample decrease significantly upon addition of IgM but not the addition of IgA. Even with addition of 50 μg/mL of IgM, which roughly doubles the concentration of IgM, we observed a significant drop in the overall IgG signals (*p* < 0.04). In contrast, the IgM signals were relatively unchanged upon the addition of IgG or IgA. Even upon addition of up to 500 μg/mL of IgG and 200 μg/mL IgA, we did not observe significant changes in the overall IgM signals (*p* > 0.4). Only about 14% of IgM signals that were at least 4-fold above the floor decreased by 2-fold or greater upon the addition of 1000 μg/mL IgG. Similarly, About 12% of IgM signals decreased by 2-fold or greater upon the addition of 400 μg/mL IgA. Taken together, these results demonstrate that many anti-glycan IgM outcompete IgG in the context of human serum.

### Effects of competition on umbilical cord and maternal serum antibody profiles

Although fetuses produce little or no IgG, fetal IgG levels and diversity in serum at birth are nearly equivalent with maternal IgGs due to active transport of maternal IgG to the fetus [33]. Maternal IgG antibodies provide critical protection for the newborn during the first few months of life. One unexpected observation over the years is that IgG antibodies of certain antigen specificities can be detected at higher levels in fetal blood than the matched maternal blood [34–36]. This phenomenon has been attributed to preferential transport of certain antibodies across the placenta [33]. Transport, however, occurs through the Brambell receptor, which recognizes the Fc portion of IgG antibodies and transports them across the placenta. Since antibody interactions with antigen occur through the complementary determining regions of the Fab portion and transport occurs via the Fc portion, it is not clear how antigen binding properties can affect transport.

In an ongoing project within our group, we have been comparing anti-glycan antibody profiles of matched cord and maternal sera. Like previous reports, we found that IgG antibodies to certain glycans were detected at higher levels in cord blood relative to maternal blood. Although preferential transport could account for this difference, we noticed that many of the “enriched” IgG antibodies recognized glycans that are also bound well by IgM. This observation led us to postulate an alternative explanation as to why certain IgG signals are higher in cord versus maternal blood. Rather than preferential transport, we hypothesized that IgG levels are approximately equivalent in cord and maternal sera and that competition with IgM in maternal blood results in reduced IgG signals. Since fetal serum has very low levels of IgM (IgM and IgA are not transported), the reduction in IgG signals only occurs in maternal blood. As IgM levels and avidities vary from antigen-to-antigen, certain IgG signals are strongly inhibited while others are not affected making it appear as though some IgG antibodies are selectively enriched in fetuses.

To test this hypothesis, we compared matched cord and maternal sera from 3 mother-baby pairs with and without depletion of IgM. We anticipated that removal of serum IgM would restore IgG binding leading to similar IgG signals in cord-maternal pairs. Cord and maternal serum samples were either treated with anti-IgM resin or control resin and then signals were compared. Samples treated with control resin had a number of antibody signals higher in cord than maternal blood. For these three serum pairs, there were a combined total of 102 IgG signals (∼10% of all glycans on the array) that were at least 4-fold higher in cord relative to maternal sera, and 33 IgG signals that were at least 10-fold higher in cord sera. Interestingly, many of the signals that were higher in cord sera were antibodies to non-human glycans (e.g. rhamnose, the Forssman antigen). More specifically, 37 of the 102 signals that were 4-fold higher in cord and 20 of the 33 signals that were 10-fold higher in cord were to non-human glycans. Adult humans have especially high IgM signals to non-human glycans, which could result in high levels of competition for this family of antibodies.

Next we evaluated IgM and IgG signals after treatment with anti-IgM resin. Depletion of IgM was verified by comparing anti-glycan IgM signals for anti-IgM resin-treated and control resin-treated samples. As expected, treatment of sera with anti-IgM resin resulted in a large decrease in all IgM signals (the median IgM signal decreases >15 fold relative to the control resin); most (>80%) glycans had no measurable IgM signal over background. Interestingly, when we compared IgG signals for cord/maternal pairs after IgM depletion, none of the cord IgG signals were >4-fold higher than maternal IgG (as compared to 102 for the control resin). The absence of differences between cord and maternal IgG profiles after IgM depletion was due to increases in various maternal IgG signals, rather than decreases in cord IgG signals. It is important to note that anti-IgM resin treatment had little to no effect on serum IgG signals for cord samples, indicating that the resin does not directly affect IgG antibodies. Since depletion of IgM abolished the “enriched” IgG signals in cord blood, we believe the observed effect is due to IgM competition, at least for the anti-glycan antibodies evaluated in this study.

## Discussion

There is growing interest in studying the biological roles of anti-glycan antibodies and exploiting this family of antibodies as biomarkers. Recently, glycan microarray technology has emerged as a powerful high-throughput tool for profiling serum anti-glycan antibodies (for recent reviews, see [26,37,38]; for some recent examples, see [27,28,39–73]). While serum contains a mixture of anti-glycan antibodies that can recognize the same antigen, the effect of competition between different serum isotypes toward printed glycans has not been studied. Such competition can influence the detection of antibody subpopulations that are relevant to diseases. Using a multiplexed microarray-based assay, we evaluated competition between IgG, IgA, and IgM anti-glycan antibodies toward a broad range of printed glycan antigens. Our results demonstrate that many IgM anti-glycan antibodies outcompete IgG and IgA antibodies toward printed glycans. The competitive advantage of anti-glycan IgM is in contrast to antibodies against other biomolecules such as proteins, where IgG antibodies are normally removed from serum to reduce interference of competing IgG in IgM or IgA tests.

Our results have important implications for studies involving detection of glycan-binding antibodies. Measurements of serum IgG and IgA anti-glycan antibodies can be significantly influenced by serum IgM levels. For example, two individuals with the same exact IgG antibody clones and serum concentrations for a particular glycan could appear to have different levels if they have differing amounts of competing IgM. Thus, it is critical to consider IgM levels when performing case-control studies to identify potential biomarkers. IgM can also influence measured changes induced by infection, disease state, or vaccination. An increase in IgM could result in a significant decrease in IgG and/or IgA signals or an underestimation of increases. Likewise, a decrease in IgM could lead to an increase in measured signals for IgG and/or IgA. For reference, total serum IgM levels in humans often vary 5–10 fold under various physiological conditions, such as hyper IgM syndromes and liver disease. Moreover, IgM titers to individual antigens often increase >10-fold after vaccination or infection. Thus, when profiling anti-glycan serum IgG or IgA antibodies, it is critical to account for IgM competition. One approach would be to eliminate competition, either by purifying the IgG or IgA prior to measurement or by removing IgM from serum prior to detection of IgG or IgA. A second approach would be to measure IgM and consider the signals, or changes in signals, when analyzing and interpreting IgG and IgA data. By accounting for IgM, one can obtain a more accurate measure of IgG and IgA.

Although the results demonstrate that IgM can affect IgG and IgA measurements for glycan-binding antibodies, certain limitations of our study should be noted. First, the molecular basis for the competitive advantage of IgM is not yet clear. There are several reasons why IgM anti-glycan antibodies outcompete IgG and IgA. Serum IgM could contain a higher proportion of glycan-binding antibodies than IgG or IgA. In this case, addition of 1 “serum equivalent” of IgM may represent a much larger amount of added competitor than addition of 1 “serum equivalent” of IgG or IgA. In our experiments, we added as much as a 16-fold excess of IgG by weight (800 μg/mL) with essentially no effect on IgM signals. Therefore, the proportion of IgM that are glycan-binders would have to be >16 times higher than the proportion of IgG glycan binders to account for the competitive advantage of IgM. Alternatively, IgM antibodies may have higher overall binding avidity toward glycan antigens than IgG and IgA. In other studies we have found that monoclonal IgM antibodies can bind very tightly to glycans on our array surface, with apparent Kd values in the low nanomolar to subnanomolar range [74,75]. Additionally, IgM may bind faster than IgG and the experimental conditions may not allow sufficient time for full equilibration. Of course, the competitive advantage of IgM may be due to a combination of these effects. Additional studies will be needed to evaluate these possibilities. Second, the competitive advantage of IgM was observed in a glycan microarray assay under a certain set of conditions. Given the similarities between the microarray assay and other assays involving surface-bound antigens, it is likely that IgM will also have a competitive advantage in other assays and under other conditions. Nevertheless, the effects of IgM competition will need to be evaluated experimentally for other cases.

In summary, we have demonstrated that anti-glycan IgM can outcompete IgG and IgA for surface-bound carbohydrate antigen in a microarray assay. Accurate measures of glycan-binding IgG and IgA are critical for studying immune responses to carbohydrate antigens and for identifying serum antibody subpopulations with utility as biomarkers. By accounting for IgM competition, one can obtain a more true measure of anti-glycan IgG and IgA levels, thus facilitating both basic research and clinical applications in the field of glyco-immunology. Finally, our results highlight the unique aspects of antibody-carbohydrate interactions and emphasize the need to consider these interactions separately from antibody-protein interactions.

## Materials and Methods

### Serum samples

Purified serum antibodies (IgG I2511, IgM I8260, and IgA I4036) and all the different IgG subclasses (IgG_1_ I5154, IgG_2_ I5404, IgG_3_ I5654, IgG_4_ I4639) were purchased from Sigma-Aldrich (St. Louis, MO). The pooled reference serum was purchased from Valley Biomedical Products and Services (Winchester, VA). Samples were tested in accordance with FDA regulations and found to be negative for HBSAG, HIV, and HCV. Sera of three matched cord-maternal pairs were purchased from ProMedDx (Norton, MA).

### Western blot

Purified serum antibodies were heat denatured in 50% Laemmli sample buffer (Bio-Rad #161-0737) and 2.5% β-mercaptoethanol. The denatured proteins and molecular weight markers were loaded into a Novex 4–20% Tris-Glycine gel. The proteins were separated by gel electrophoresis using 1X NuPAGE MES SDS running buffer at constant voltage (125 V) for 1.5 hrs. The separated proteins were transferred to nitrocellulose membrane and blocked with StartingBlock blocking buffer (Thermo Scientific 37538) for 2hrs. The membrane was rinsed 3 times with PBST (PBS with 0.05% (v/v) Tween 20) and then incubated for 1hr with the proper goat anti-human detection reagent (Jackson ImmunoResearch, DyLight 549 anti-human IgG: 109-505-008, DyLight 649 anti-human IgM: 108-495-043, DyLight 549 anti-human IgA: 109-506-011) diluted 1:500 in the blocking buffer. The membrane was washed 6 times with PBST and imaged with ImageQuant LAS 400.

### Dot blot assay

The diluted working concentrations from all samples were prepared in phosphate-buffered saline (PBS; pH 7.4). From the diluted solutions 2.0 μL were spotted in duplicate onto a nitrocellulose membrane and were allowed to dry at room temperature. The membrane was block with StartingBlock blocking buffer for 2hrs. The membrane was rinsed 3 times with PBST and then incubated for 1hr with the proper goat anti-human detection reagent (Jackson ImmunoResearch, DyLight 549 anti-human IgG: 109-505-008) diluted 1:500 in the blocking buffer. The membrane was washed 6 times with PBST, imaged with ImageQuant LAS 400, and analyzed with Genepix Pro 6.0 software to obtain the fluorescence intensity signals.

### Array fabrication and binding assay

Glycan arrays were fabricated as previously reported [74] except for the addition of a washable fluorescent dye to the print buffer as an indicator of successful liquid deposition and spot morphology (see S1 File). The array format and assay have been described previously [76], along with analysis of reproducibility [77] and validation with numerous antibodies and lectins [78–81]. Please see the Supplemental Glycan Microarray Data (S2 File) for details on the carrier proteins, conjugation methods, and densities of glycans conjugated to the carriers. Prior to each experiment, slides were pre-scanned (GenePix 4000B, Molecular Devices) and then a 16-well module (Grace Bio-Lab) was affixed to the slide in order to create 16 independent array wells. Pre-scanned images were analyzed for technical faults and saved as a permanent record. The slides were blocked overnight at 4°C with 3% BSA (w/v) in PBS and washed 6 times with PBST. Samples were diluted in 3% BSA and 1% HSA in PBST, and then 100 μL of each sample was added into two different wells on different slides and allowed to incubate at 37°C with gentle agitating (100 RPM) for 4 h. After washing 3 times with PBST, bound antibodies were detected by incubating with DyLight labeled goat anti-human detection reagents (IgG, IgA, or IgM; 3 μg/mL) in 3% HSA and 1% BSA in PBS for 2 h at 37°C with gentle agitating. After washing 7 times with PBST, slides were removed from modules, immersed in wash buffer for 5 min, and centrifuged at 1000 rpm for 5 min.

### Image analysis and data processing

Slides were scanned at 10 μm resolution with a Genepix 4000B microarray scanner (Molecular Devices Corporation) and analyzed with Genepix Pro 6.0 software as previously reported [82]. The spots were defined as circular features with a diameter of 80 μm. The background-corrected median was used for data analysis, and technical faults (e.g., missing spots) were flagged and excluded from further analysis. To minimize the impact of noise on our comparisons, spots with intensity lower than 150 RFU (1/2 the typical background signal when analyzing IgM at 1:50) were considered too low to be measured accurately and were set to 150. The average of duplicate spots was calculated and normalized to the reference samples. A log-transformed (base 2) was applied for each slide, and the final data value was obtained from the normalized average of data from both slides.

### IgM depletion

Anti-human IgM agarose (Sigma A9935) was used for IgM depletion and Sepharose 4B (Sigma, 4B200) was used as control resin. Both resins were equilibrated on column cartridges, washed with 5× PBS buffer and then mixed with equal column volume of PBS buffer. For IgM depletion assays, 110 uL anti-human IgM agarose solution was mixed with 110 uL cord or maternal serum samples (4× dilution, in PBS buffer containing 3% BSA, 1% HSA and 0.05% Tween 20, pH 7.4). In the control assays, 110 uL Sepharose 4B was mixed with 110 uL diluted sera. These solutions were incubated at room temperature for 1.5 h with gentle agitation (150 rpm). The resins were centrifuged at 10,000×g for 5 min, and the serum supernatant (∼130 μL/sample) was collected for antibody profiling the glycan microarray. Both cord and maternal sera were treated using the same method.

Each sample was evaluated on a glycan microarray in duplicate as described above with the following differences: the volume of serum samples was 60 μL/well, the slides were scanned with a GenePix 4000B fluorescent scanner (Molecular Devices), and fluorescent intensity for each array component was quantified using GenePix Pro 7.0 software (Molecular Devices). Since these samples were evaluated at a dilution of 1:8, any signals lower than 900 RFU were adjusted to 900 (floor).

## Supporting Information

S1 File Supporting experiments, tables, and figures. (DOCX)

S2 File Supplemental Glycan Microarray Data. (XLSX)
