# Supplementary material for: Competition between Serum IgG, IgM, and IgA Anti-Glycan Antibodies
Source: PLoS One. 2015 Mar 25;10(3):e0119298. doi: 10.1371/journal.pone.0119298 (PMC4373866; doi:10.1371/journal.pone.0119298)
Supplement: S1 File — (DOCX) [file pone.0119298.s001.docx]

**Competition between Serum IgG, IgM, and IgA Anti-Glycan Antibodies**

**Saddam M. Muthana, Li Xia, Christopher T. Campbell, Yalong Zhang, and Jeffrey C. Gildersleeve**

Chemical Biology Laboratory, National Cancer Institute, NIH

376 Boyles St, Frederick, MD, 21702, USA

**Table of Contents Page**

[Quality control measures for printed arrays 2](#_Toc315873376)

Characterization of detection reagents 3

Multiplexed assay for simultaneous detection 4

[Figure A 7](#_Toc315873376)

Figure B 8

Figure C 9

[Figure D 10](#_Toc315873377)

Figure E 11

Figure F 12

[Figure G](#_Toc315873377) 13

Figure H 14

Figure I 15

[Figure J](#_Toc315873377) 16

**Quality control measures for printed arrays**

Our arrays are produced by printing neoglycoproteins (glycoconjugates containing glycans covalently attached to a carrier protein via a non-native linkage) onto epoxide-coated glass microscope slides (ArrayIt) using a robotic arrayer (MicroGrid II). We had previously inspected microarrays manually under a microscope to identify technical faults, such as missing, merged, or smeared spots; however, this approach was slow, laborious, and error-prone. We experimented with adding a commercially available fluorescent dye to the print buffer as an indicator of successful liquid deposition and spot morphology. We sought a dye that (1) would not interfere with the printing of neoglycoproteins, (2) could be detected using the same fluorescent scanner used to analyze processed slides, (3) would be completely removed during the normal blocking and washing of arrays during a typical experiment, and (4) would be stable for hours to days in aqueous solution at room temperature during printing of microarrays and for months when arrays are stored at −20 °C.

After considering a variety of dyes, the free acid of DyLight 649 was found to meet all of criteria. We found that we could print symmetric spots with a diameter of 80-100 µm when a water-soluble fluorescent dye was included in the print buffer (0.2-1.0 µg/mL). Pre-scanning a slide to identify missing and merged spots could be completed in 8 minutes. Although some decrease in signal occurred when slides were left exposed during extended print runs (48 hours), this gradual fading of fluorescence could be reduced by protecting the slides from light during printing. The fluorescent dye also remained stable during long-term storage (at least 1 year) of array slides in the dark at −20 °C. Importantly, the dye was completely removed after our standard wash cycle. This is critical, as residual dye would create fluorescent background. Therefore, adding a water-soluble fluorescent dye to the print buffer is a convenient way to replace visual inspection of microarrays for technical faults, and it requires minimal effort, expense, and no additional equipment. In addition to the time savings, pre-scanning slides provides a permanent record of slide quality.

**Characterization of detection reagents**

Labeled secondary antibodies are commonly used for the detection of serum antibodies, but characterization of these commercial reagents is often lacking. Without independent verification of the specificities of detection reagents, wrong conclusions may be drawn. We selected goat anti-human secondaries (IgA, IgG, and IgM) labeled with either DyLight 549 or DyLight 649. These particular secondary antibodies were chosen for their high specificity and minimal cross-reactivity, and we have been using them for profiling serum anti-glycan antibodies. Using purified IgA, IgG, and IgM antibodies (Sigma-Aldrich, St. Louis, MO) from human pooled serum, we evaluated the specificity of secondary antibodies using SDS-PAGE gels and western blots (Figure S1). We found that these secondary antibodies have no cross-reactivity with other isotypes. These secondary antibodies reacted only with the heavy chain portions of the corresponding antibodies with no observed reactivity against the light chains.

Next, we evaluated the specificity of the IgG secondary towards different IgG subclasses (IgG_1_-IgG_4_). Since the IgG secondary react with the Fc portion of the heavy chain, variations in the light chains should have minimum impact on the detection of different IgG subclasses. Different concentrations (25µg/mL to 800µg/mL) of all the IgG subclasses (IgG_1_, IgG_2_, IgG_3_, IgG_4_; Sigma-Aldrich, St. Louis, MO) were prepared in phosphate-buffered saline (PBS; pH 7.4). From the diluted solutions 2.0 µL were spotted in duplicate onto nitrocellulose membrane and were allowed to dry at room temperature. The membrane was block with StartingBlock blocking buffer (Thermo Scientific 37538) for 2hrs. The membrane was rinsed 3 times with PBST (PBS with 0.1% (v/v) Tween 20) and then incubated for 1hr with DyLight 549 goat anti-human IgG diluted 1:500 in blocking buffer. The membrane was washed 6 times with PBST, imaged with ImageQuant LAS 400, and analyzed with Genepix Pro 6.0 software. As expected, the IgG secondary reacts with all the different IgG subclasses (Figure S2). In comparison to serum IgG antibodies, the cross-reactivity of the IgG secondary toward different IgG subclasses were 104% for IgG1, 98% for IgG2, 90% for IgG3, and 74% for IgG4.

**Multiplexed assay for simultaneous detection**

Previously, we measured levels of serum anti-glycan antibodies using a single secondary antibody in each experiment, or using secondary antibodies that are conjugated to the same fluorophore to obtain total levels. One strategy to improve throughput and obtain more information from each array experiment is to combine differentially labeled secondary antibodies specific for different antibody isotypes (e.g., IgG and IgM). A dual channel scanner can then independently detect these two secondary antibodies in a single experiment. Although this approach is simple conceptually, a number of potential technical problems can arise. In particular, one must ensure that the mixture of dyes and secondary reagents do not interfere with each other and that the mixture provides equivalent information as assaying each secondary independently.

For multiplexing detection, we selected goat anti-human IgG and IgM secondaries that were labeled with DyLight 549 and DyLight 649, respectively. These particular secondary antibodies were chosen for their high specificity and minimal cross-reactivity to other isotypes as described above. The IgG secondary (Jackson ImmunoResearch 109-505-008) reacts with the Fc portion of human IgG heavy chain but not with human IgM, IgA, or non-Ig serum proteins. The IgM secondary (Jackson ImmunoResearch 109-495-043) reacts with the Fc_5µ_ portion of the human IgM heavy chain but not with human IgG, IgA, Ig light chains, or non-Ig serum proteins. The relative signals obtain using the IgM secondary were consistently 20-30% higher than that of IgG (Figure S3).

To verify compatibility of the secondary antibodies, we first assessed the equivalency of data obtained with the same secondary antibody conjugated to different fluorophores. Data obtained with secondaries conjugated to DyLight 549 and DyLight 649 were highly correlated (Figure S4). Array signals were consistently higher when using secondary antibodies conjugated to DyLight 649 compared to array signals measured using DyLight 549 conjugated secondaries. However, because data collected with different fluorophores was linearly correlated, these data can be directly compared after applying an appropriate normalization technique, such as with median centering (proportionally scaling data so that the adjusted median of the reference sample is equal on all slides).

To obtain data independently from two secondary antibodies, it is critical that each dye only produces signal in the appropriate channel (i.e. no bleed-through to the other channel) and that there is no cross-talk between the fluorophores through mechanisms such as fluorescent resonance energy transfer (FRET). DyLight 549-labeled goat anti-human IgG and DyLight 649-labeled anti-human IgM were found to have almost no bleed-through across the red and green channels (Figure S5). In addition, data collected using a single secondary antibody was nearly identical with data collected in the presence of both secondary reagents (Figure S6). Taken together, these experiments demonstrate that IgG and IgM can be reliably detected in a single experiment.


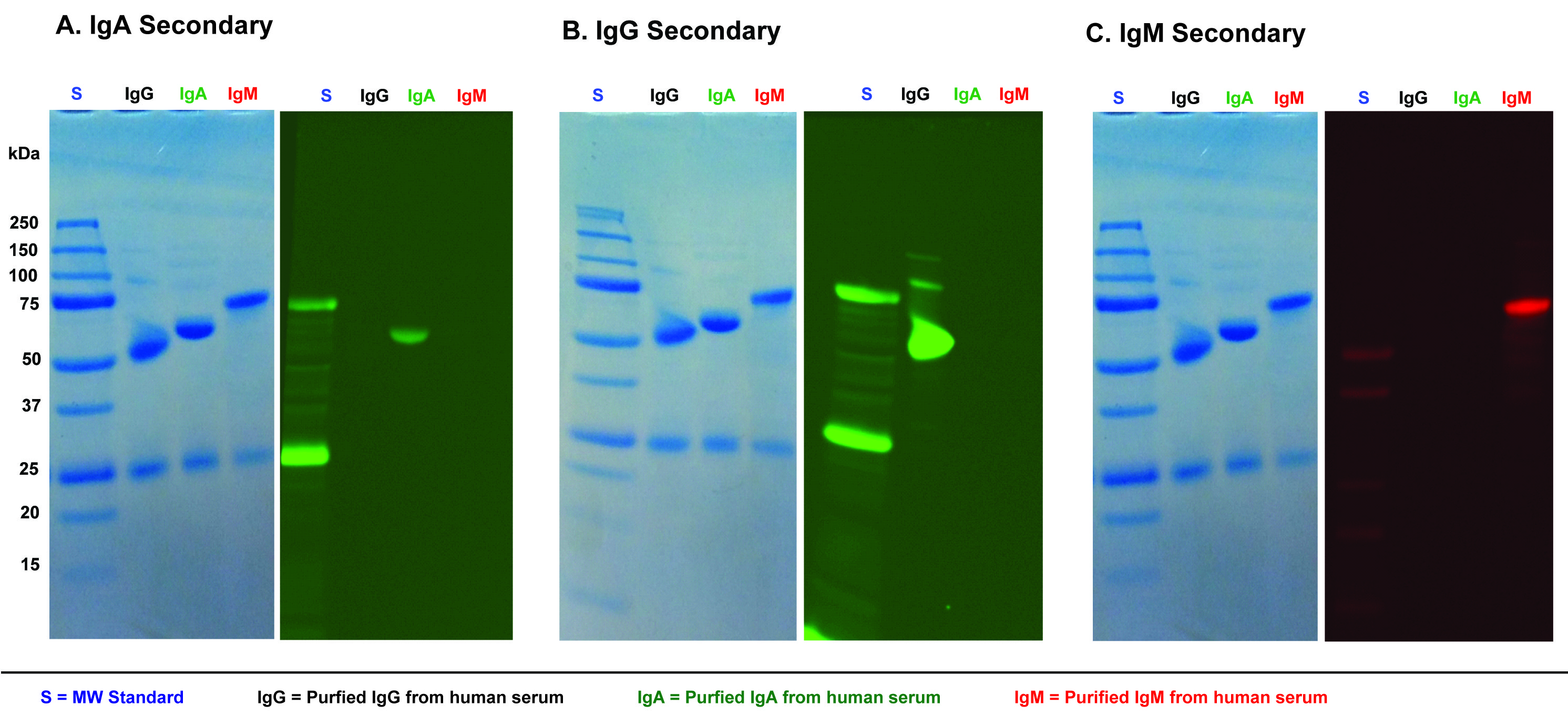


**Figure A**. **SDS-PAGE gels of purified antibodies from human serum under reduced condition and western blots after incubation with secondary antibodies**. (A) DyLight 549 goat anti-human IgA (Jackson ImmunoResearch 109-506-011), (B) DyLight 549 goat anti-human IgG (Jackson ImmunoResearch 109-505-008), and (C) DyLight 649 goat anti-human IgM (Jackson ImmunoResearch 109-495-043).


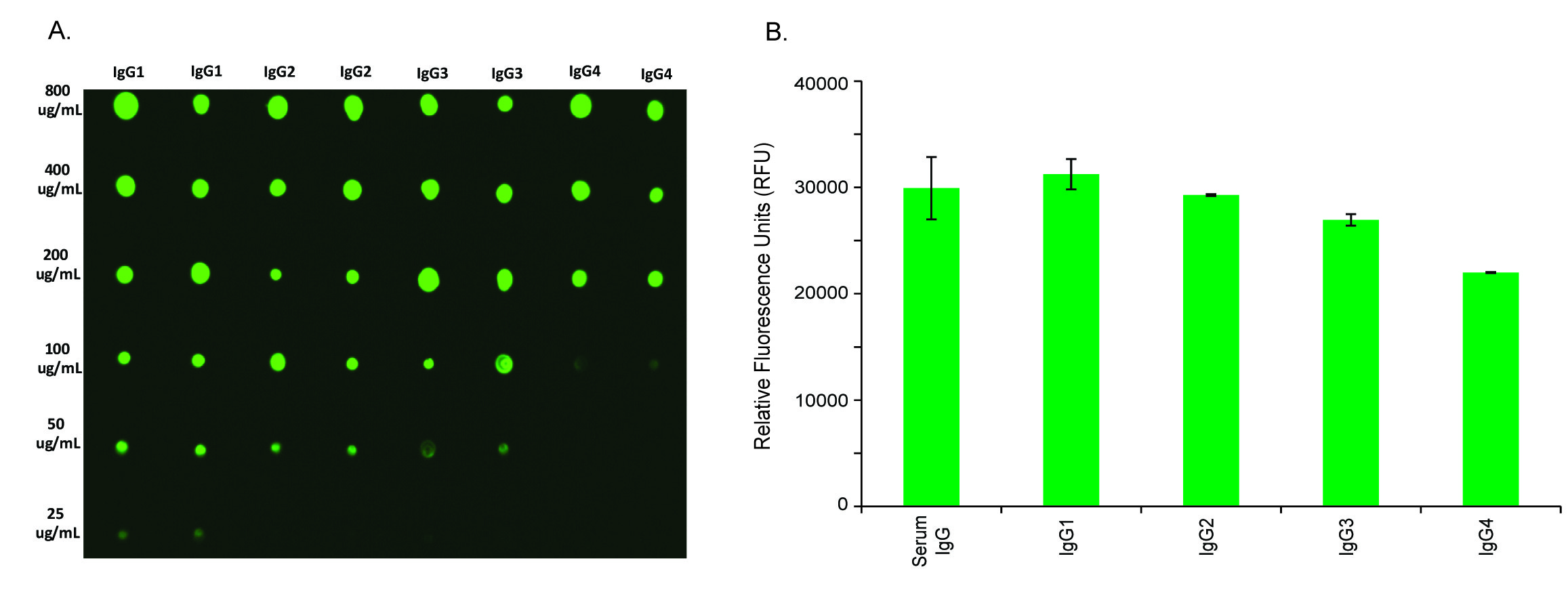


**Figure B**. **Reactivity of IgG secondary toward different IgG subclasses**. (A) A representative image of a dot blot detecting different IgG subclasses using DyLight 549 goat anti-human IgG (Jackson ImmunoResearch 109-505-008). (B) Fluorescence intensities of spotted serum IgG (diluted 1:50) and different IgG subclasses (200µg/mL).


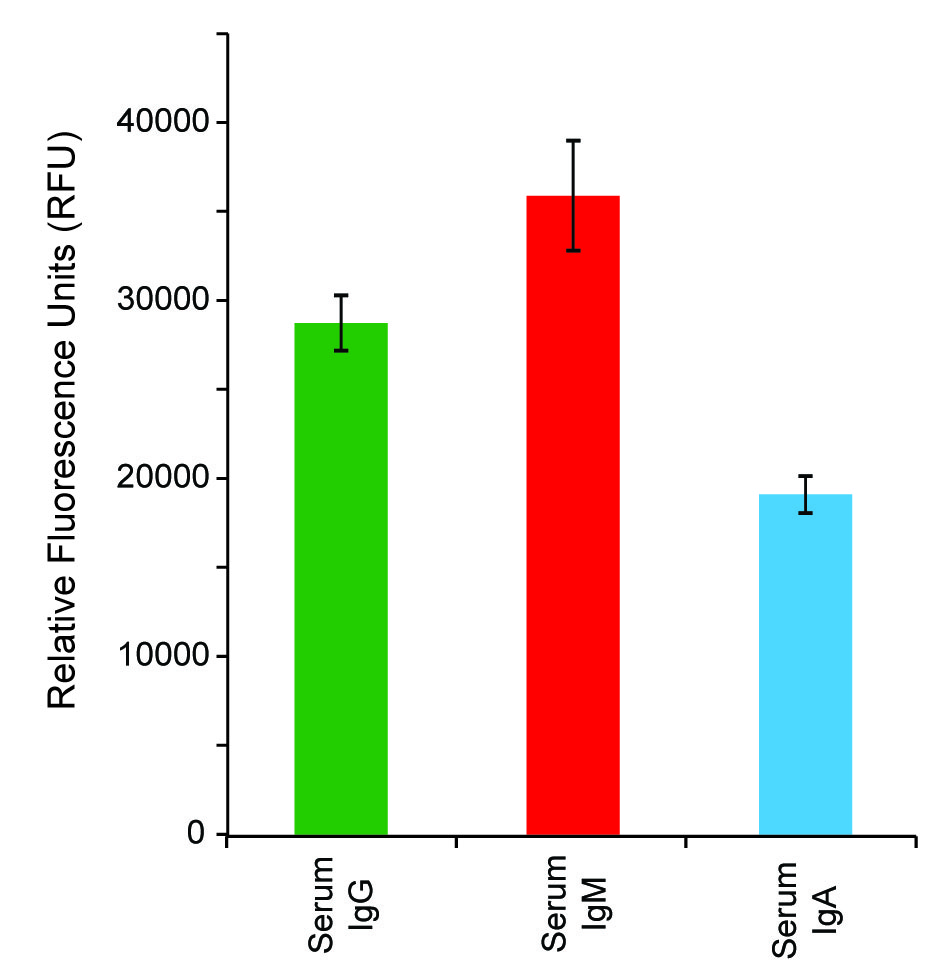


**Figure C**. **Relative signals and reactivity of IgG, IgM, and IgA detection antibodies**. Signals measured using IgM secondary were consistently 20-30 % and 45-55% higher than that of IgG and IgA, respectively.


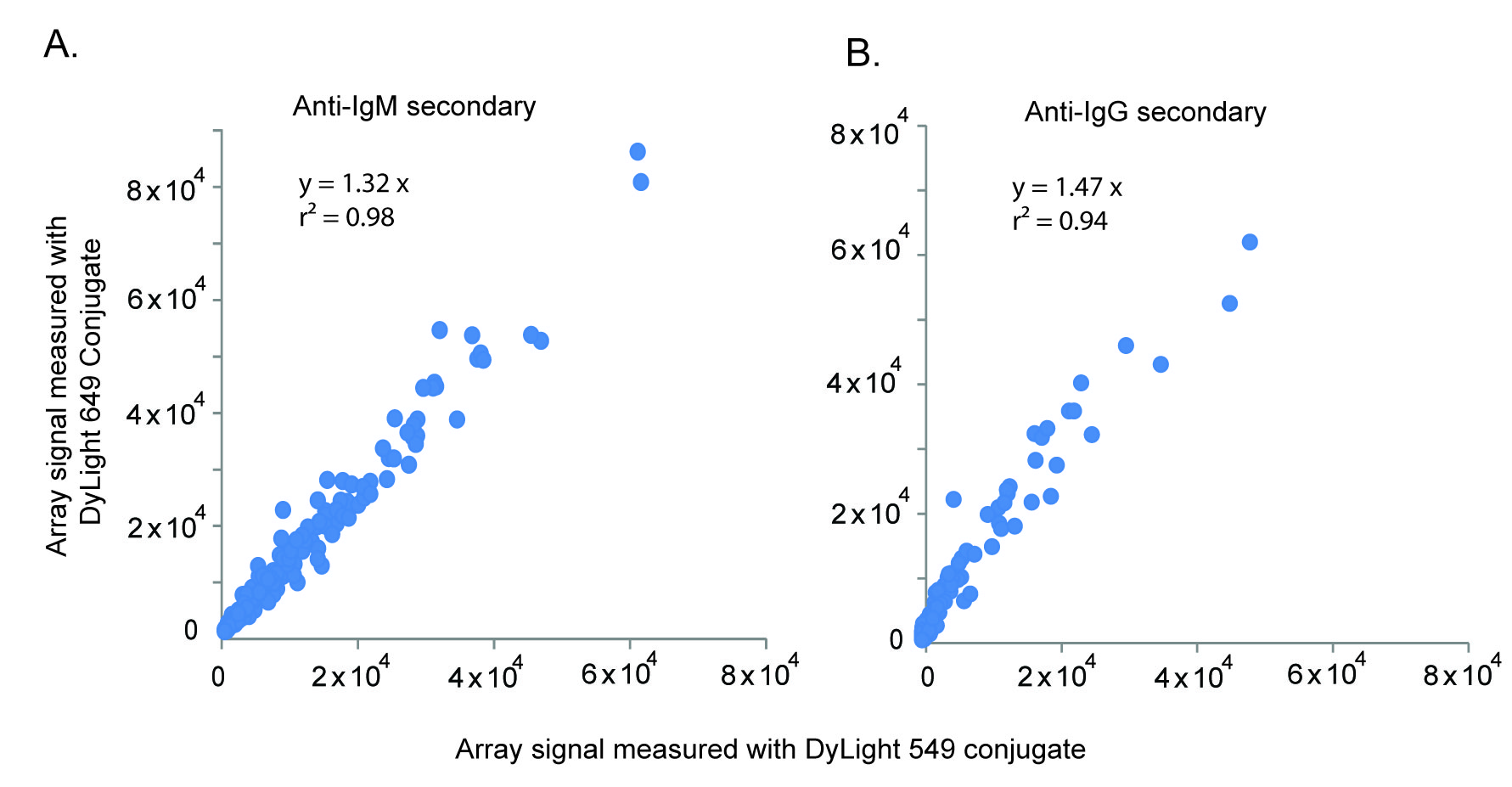


**Figure D**. **Effect of fluorophore on measurements of serum anti-glycan antibody levels**. (A) Levels of antibodies were assayed with the same secondary antibody (A = anti-IgM; B = anti-IgG) conjugated to different fluorescent dyes (DyLight 649 or DyLight 549).


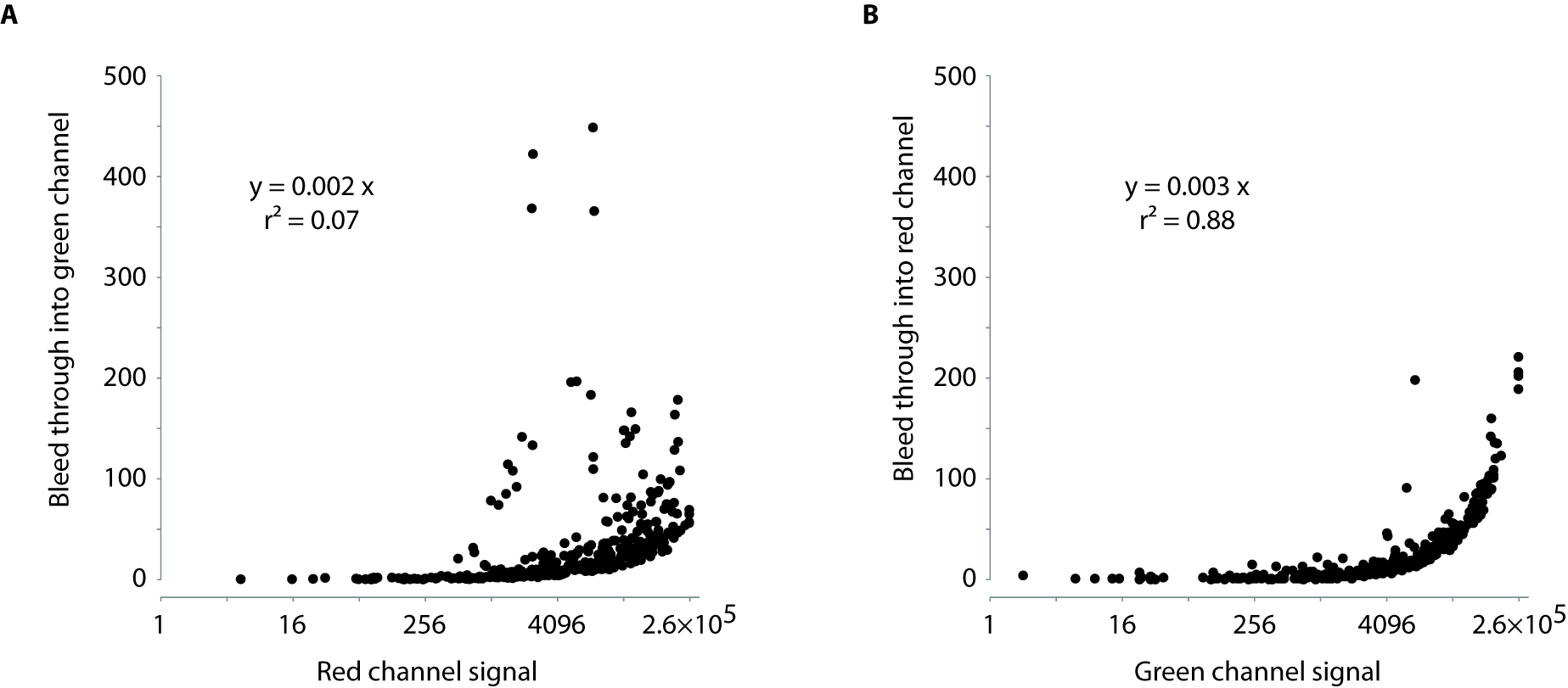


**Figure E**. **Bleedthrough across red and green channels**. Fluorescence signals were measured simultaneously in the red and green channels when single secondary antibodies were used individually. (A) Fluorescence intensity measured in two channels when using a DyLight 649 conjugated secondary antibody. (B) Similarly, fluorescence intensity measured in two channels for a DyLight 549 conjugated secondary antibody. Bleedthrough across channels (y axes) was minimal and correlated with signal in the primary channel (x axes). To visualize bleedthrough, the y-axis is on the linear scale while the x-axis is in log base 2.


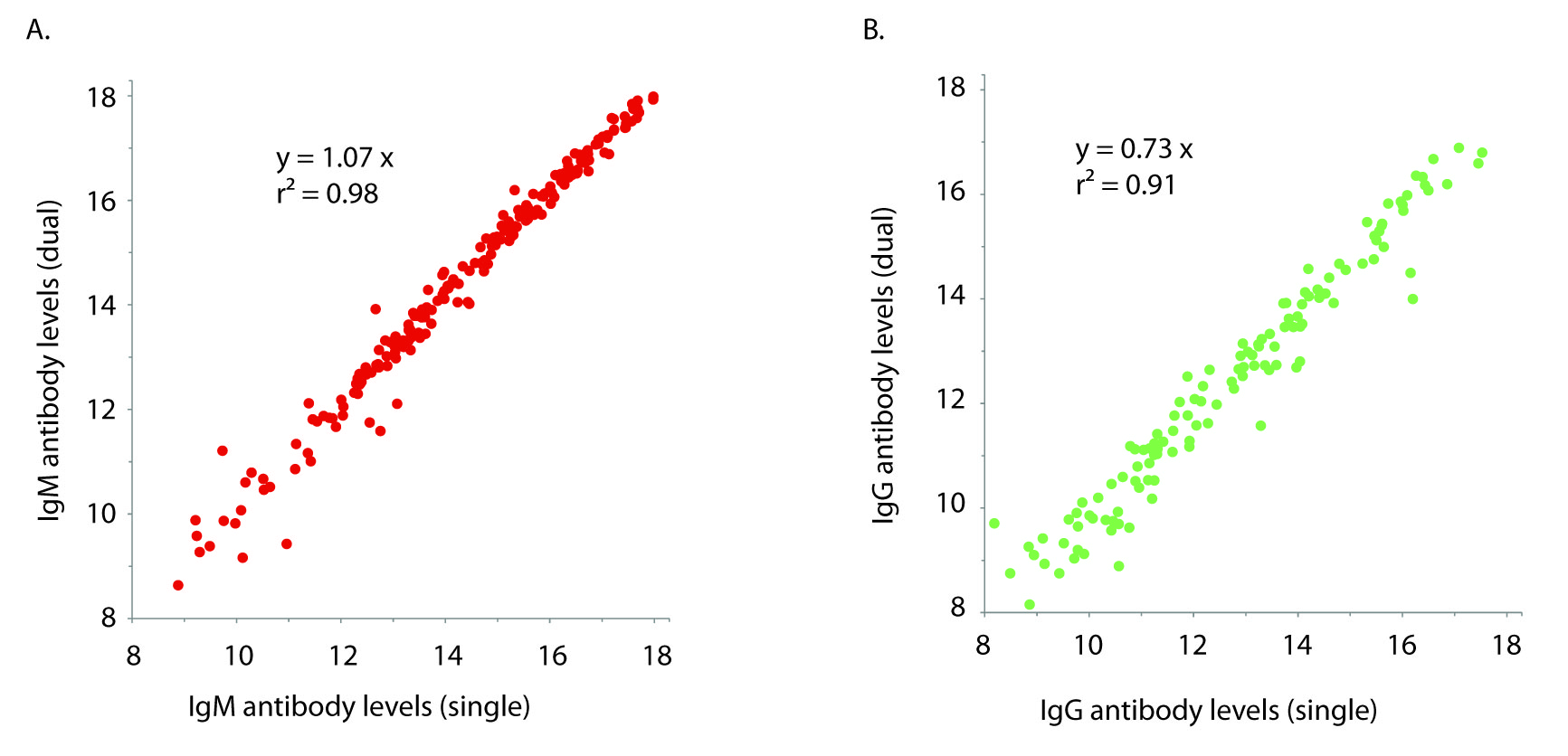


**Figure F**. **Levels (log-transformed base 2) of serum anti-glycan antibodies measured with single or dual secondary antibodies**. Microarray profiling was performed using a single secondary antibody (anti-IgM or anti-IgG) or a combination of differentially labeled secondaries (DyLight 649 and DyLight 549). (A) IgM levels measured using only DyLight 649 anti-IgM antibody (x axes) were plotted against IgM levels measured with the same anti-IgM secondary antibody in the presence of DyLight 549 anti-IgG. (B) Similarly, IgG levels were measured with DyLight 549 anti-IgG alone (x axes) or in the presence of differentially labeled DyLight 649 anti-IgM (y axes).


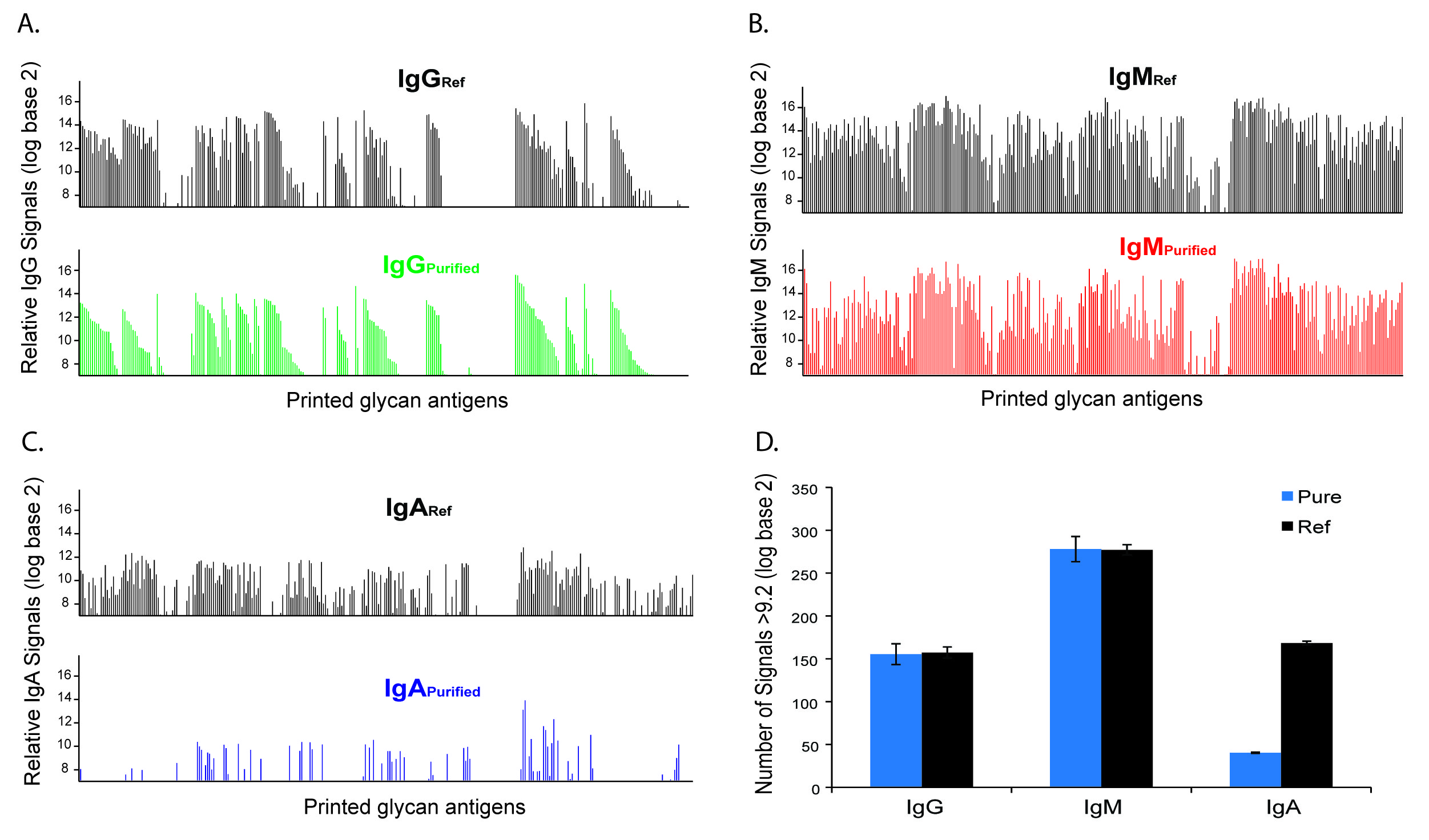


**Figure G..** **Distribution of anti-glycan antibodies (IgG, IgM, and IgA)**. (A) IgG signals in reference and purified sample. (B) IgM signals in reference and purified sample. (C) IgA signals in reference and purified sample. (D) Number of IgG, IgM, and IgA signals in reference serum (1:50 dilution) and purified antibodies (200 μg/mL IgG, 50 μg/mL IgM and 50 μg/mL IgA) that are 4-fold above the set floor (150 RFU).


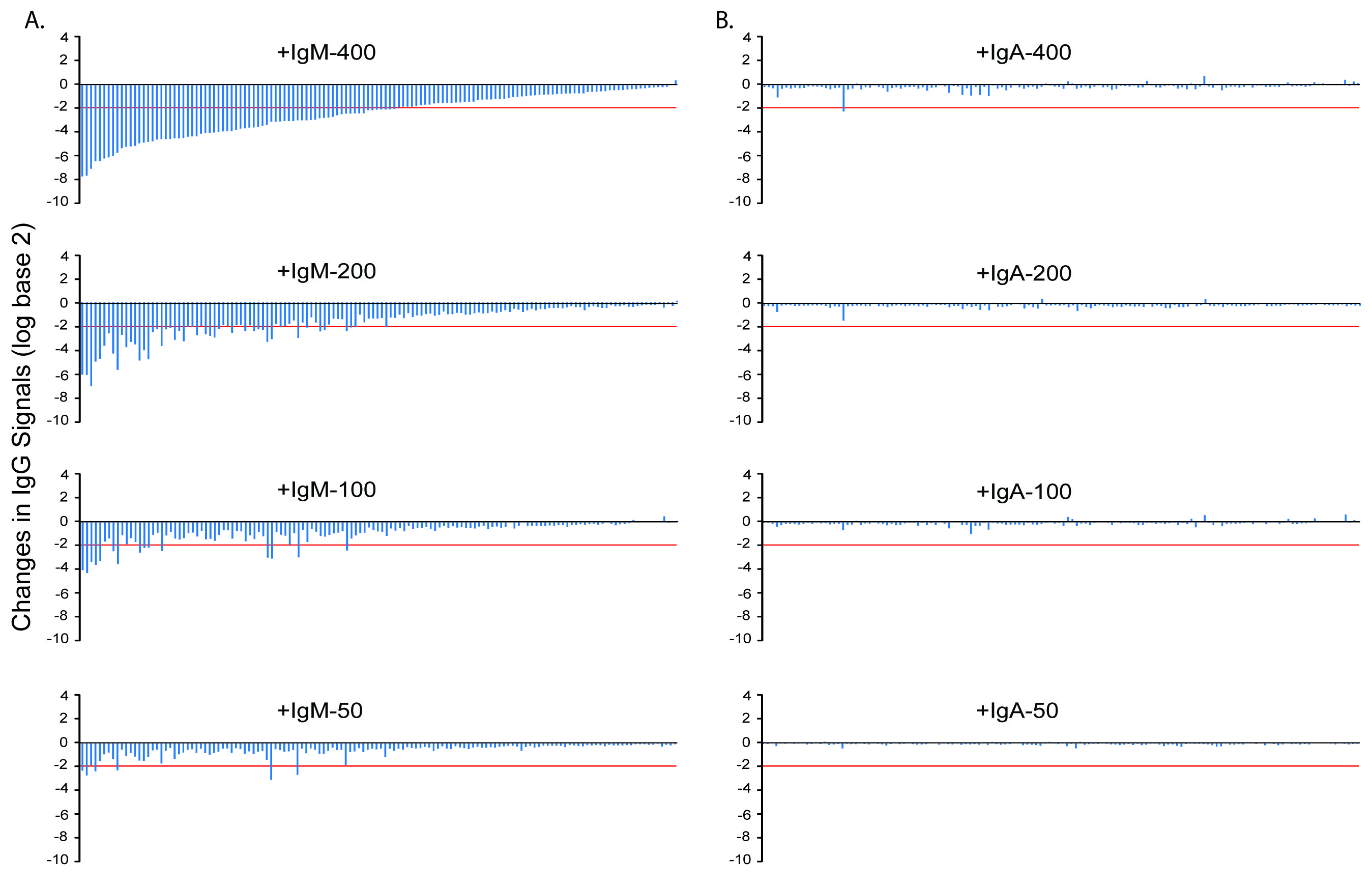


**Figure H**. Changes in IgG signals upon increasing the concentration of IgM (A) and IgA (B). The x-axis contains the printed glycans with IgG signals.


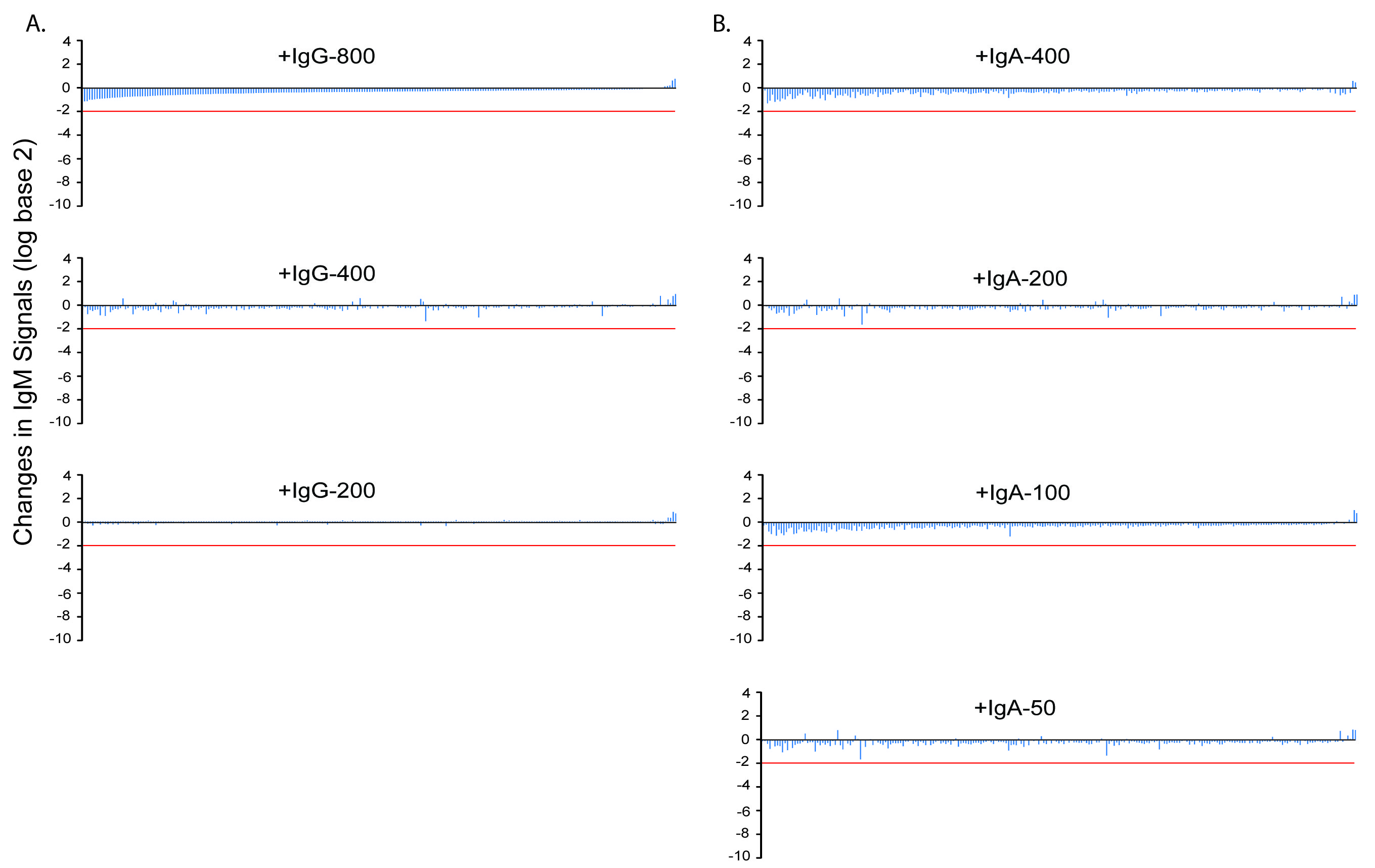


**Figure I**. Changes in IgM signals upon increasing the concentration of IgG (A) and IgA (B). The x-axis contains the printed glycans with IgM signals.


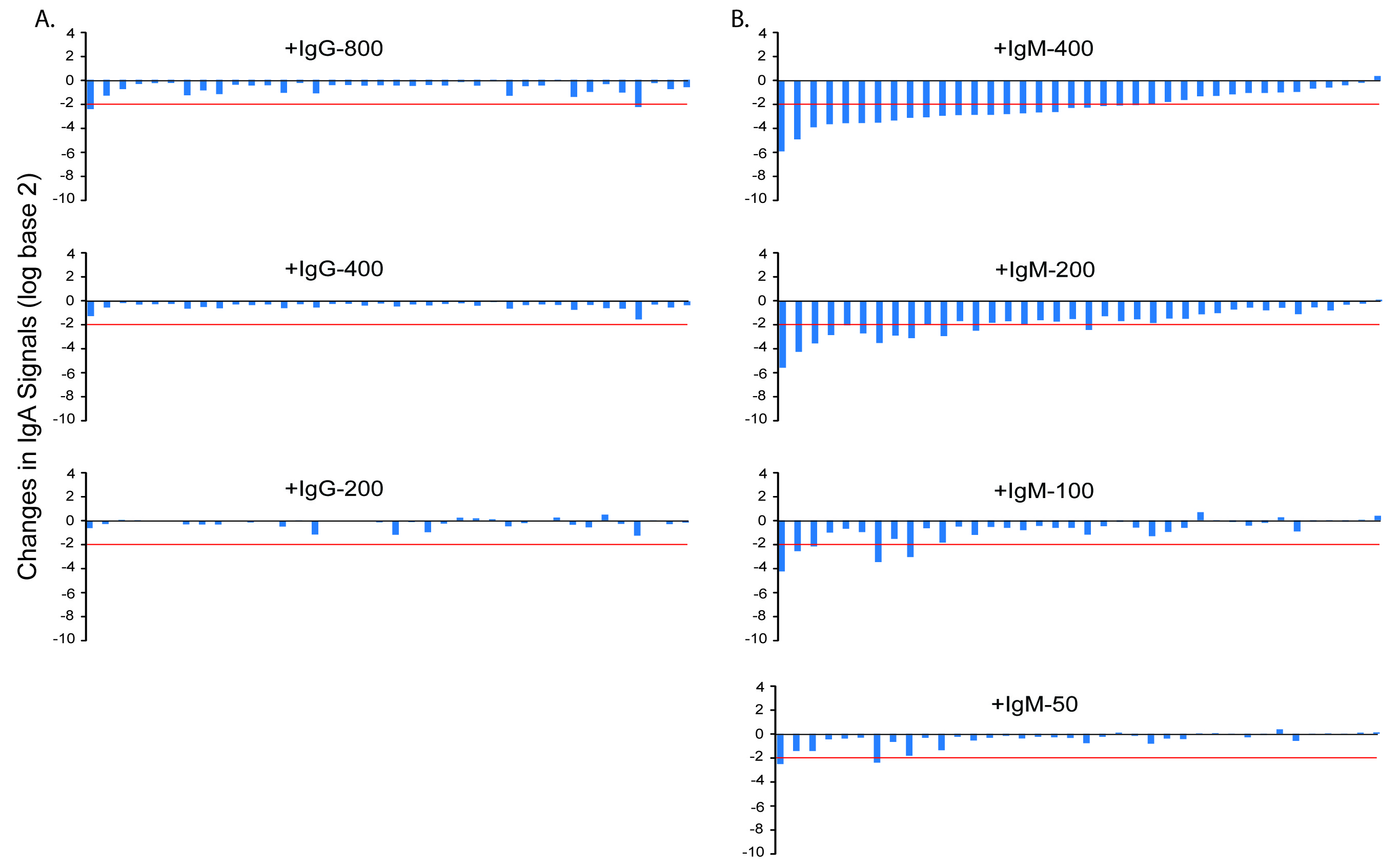


**Figure J**. Changes in IgA signals upon increasing the concentration of IgG (A) and IgM (B). The x-axis contains the printed glycans with IgA signals.
